# An approach towards next-generation hydrogen storage: a DFT study on A_2_LiTiH_6_ (A = K, Ca) perovskite hydrides

**DOI:** 10.1039/d5ra04660b

**Published:** 2025-10-15

**Authors:** Muhammad Abaid Ullah, Muhammad Kaleem, Amna Nasir, Zahid Sarfraz, Malik Muhammad Asif Iqbal, Muhammad Rizwan, Khalid Nadeem Riaz, Muhammad Tanzeel

**Affiliations:** a Department of Physics, University of Okara Pakistan abaidullah91@gmail.com; b Department of Physics, International Islamic University H-10 Islamabad 44000 Pakistan mkaleemphy@gmail.com; c Department of Physics, Air University, E9 Islamabad 44000 Pakistan; d Department of Chemistry, University of Okara Pakistan; e School of Physical Sciences, University of the Punjab Lahore Pakistan

## Abstract

This study explores the structural, mechanical, hydrogen storage, optical and thermodynamic properties of the double perovskite hydride A_2_LiTiH_6_ (A = K, Ca) by means of density functional theory (DFT). With tolerance factors of 0.997 for K_2_LiTiH_6_ and 0.903 for Ca_2_LiTiH_6_, both compounds have a stable cubic *Fm*-3*m* symmetry. K_2_LiTiH_6_ and Ca_2_LiTiH_6_ have calculated formation energies of −1.182 eV and −1.037 eV, respectively, suggesting a favorable thermodynamic stability. K_2_LiTiH_6_ exhibits a gravimetric capacity of 4.38 wt% and a volumetric capacity of 19.12 g L^−1^, while Ca_2_LiTiH_6_ exhibits a gravimetric capacity of 4.29 wt% and a volumetric capacity of 23.41 g L^−1^. The desorption temperatures for K_2_LiTiH_6_ are 435.8 K and 380.4 K for Ca_2_LiTiH_6_, making both materials suitable for hydrogen release at moderately high temperatures. The mechanical analysis of both compounds showed that they are both mechanically stable, with moderate hardness (9.64–17.10 GPa) and brittleness (*B*/*G* ratios of 1.29 for K_2_LiTiH_6_ and 1.37 for Ca_2_LiTiH_6_). Electronic properties of both materials display metallic behavior, suggesting potential applications in optoelectronics. Furthermore, thermodynamic properties, such as Debye temperatures (447.2 K for K_2_LiTiH_6_ and 584.0 K for Ca_2_LiTiH_6_) and melting points (811.2 K for K_2_LiTiH_6_ and 1195.2 K for Ca_2_LiTiH_6_), indicate the robustness of these materials for practical hydrogen storage applications. In this comprehensive study, A_2_LiTiH_6_ (A = K, Ca) perovskite hydrides are identified as potentially viable candidates for hydrogen storage systems and energy harvesting technologies.

## Abstract 2

DFT-based study establishes A_2_LiTiH_6_ (A = K, Ca) double perovskite hydrides as thermodynamically stable candidates for solid-state hydrogen storage applications.

## Graphical abstract

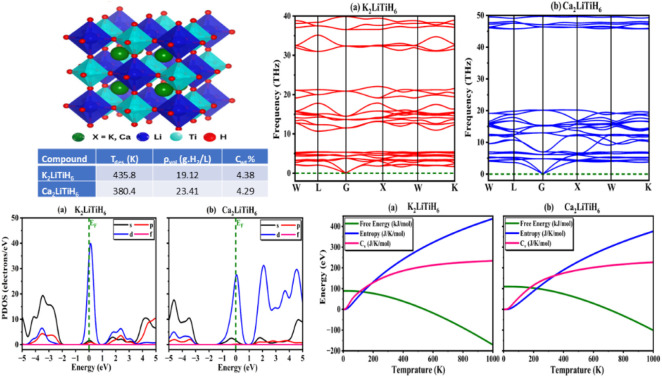

## 1. Introduction

Globally, renewable and low-carbon energy solutions are gaining momentum due to the urgent challenges posed by climate change and energy insecurity. Among renewable energy carriers, hydrogen has garnered considerable attention due to its high energy density, wide availability, and potential to drive decarbonization in various sectors, including transportation, electricity, and industry.^1,2^ Projections by the International Renewable Energy Agency (IRENA) indicate that hydrogen could contribute up to 12% of the global energy supply by 2050, positioning it as a critical pillar of future-oriented clean energy technologies.^3^ Despite this potential, the adoption of hydrogen remains constrained by fundamental challenges in storage. Conventional hydrogen storage methods, including compressed gas, cryogenic liquids, and chemical hydrides, face inherent drawbacks such as low volumetric density, safety hazards, and high energy costs. These limitations have driven increasing interest in solid-state hydrogen storage materials, which offer a promising pathway toward safe, reversible, and high-capacity hydrogen storage for a sustainable hydrogen economy.^4,5^

Among the most promising alternatives for solid-state hydrogen storage are complex metal hydrides, particularly those adopting perovskite structures, due to their high volumetric hydrogen densities (>150 kg H_2_ per m^3^), structural flexibility, and tunable thermodynamic properties.^6^ Double perovskite (DP) hydrides, with the general formula A_2_BXH_6_, feature robust three-dimensional frameworks composed of corner-sharing BX_6_ octahedra that facilitate reversible hydrogen incorporation and diffusion.^7^ A particular interest has been directed to LiAH_3_-type perovskite hydrides due to their lightweight nature and promising hydrogen storage capacity. Recent studies^8,9^ have reported the structural stability, moderate formation enthalpies, and hydrogen storage capacities of these materials, but also highlighted challenges such as limited reversibility and relatively high desorption temperatures. Based on these characteristics, it is important to investigate alternative Li- and Ti-based double perovskite hydrides, such as A_2_LiTiH_6_, to overcome these limitations. Their inherent chemical flexibility enables strategic cation substitution at both the A and B sites, providing a versatile platform for optimizing gravimetric capacity, formation enthalpy, and band structure.^8^ Several members of this family, such as NaMgH_3_,^9,10^ have demonstrated favorable thermodynamic stability, while systems including (Rb/Cs)BH_3_ (ref. 11) and Cs_2_XGaH_6_ (ref. 12) exhibit moderate hydrogen storage capacities approaching 2 wt%. Despite these advancements, most previous studies have focused on heavier alkali or alkaline-earth elements, leaving the potential of lighter, more earth-abundant cations largely unexplored.^13^ Recent computational advances have further highlighted the potential of double perovskite hydrides for applications in hydrogen storage. For example, Waqar Azeem *et al.* reported semi-metallic X_2_CaCdH_6_ (X = Rb, Cs) hydrides with indirect electronic band gaps of 2.13 and 2.30 eV, and gravimetric hydrogen storing capacities of 1.69 wt% and 1.39 wt%, respectively, supported by favorable thermodynamic stability.^14^ Similarly, Othman Hakami *et al.* investigated that A_2_FeH_6_ (A = Be, Mg) hydrides exhibit considerably higher gravimetric hydrogen storing capacities of 7.50 wt% and 5.43 wt%, respectively, along with promising mechanical and thermoelectric properties.^15^ These studies collectively emphasize the critical role of compositional engineering in optimizing hydrogen storage performance.

Despite these advances, a significant research gap persists in the targeted exploration of lightweight double perovskite hydrides,^16^ particularly those incorporating lithium (Li^+^) and titanium (Ti^3+^) at the B- and X-sites. These elements, both earth-abundant and environmentally benign, offer the distinct advantage of low atomic masses (Li: 6.94; Ti: 47.86 amu), contributing directly to enhanced gravimetric hydrogen capacities, an essential parameter for meeting the U.S. Department of Energy (DOE) targets of ≥5.5 wt% for viable hydrogen storage systems.^17^ Moreover, the unique electropositive character of Li^+^, combined with the robust Ti–H bonding framework, is anticipated to facilitate favorable thermodynamic properties, ensuring reversible hydrogen release at moderate desorption temperatures.^18^

In this framework, A_2_LiTiH_6_ (A = K, Ca) compounds represent an unexplored class of lightweight double perovskite hydrides with promising potential for achieving a balance between high hydrogen content, thermal stability, and favorable electronic characteristics. Despite their theoretical promise, comprehensive DFT-based investigations into the structural, mechanical, and hydrogen-storing attributes of these compounds underscore an urgent need for targeted Investigation. This study seeks to address that gap by engaging a principal approach within the GGA-PBE framework to systematically examine the structural, mechanical, electronic, optical, thermodynamic, and hydrogen storing characteristics of A_2_LiTiH_6_ (A = K, Ca), by elucidating the effects of A-site substitution on formation energetics, hydrogen release profiles, and mechanical behavior. This study provides essential insight into the viability of these hydrides as candidates for the implication in the field of hydrogen storage. These findings contribute to the multidimensional pursuit of sustainable energy solutions through the targeted development of next-generation hydrogen storage compounds.

## 2. Computational methodology

The ground state properties and geometry optimization were determined through DFT calculations using the CASTEP code. This approach involves solving the Kohn–Sham equations, utilizing a plane wave basis to represent the wave functions of valence electrons with kinetic energy lower than the predefined cutoff energy.^19,20^ To address exchange-correlation effects, the generalized gradient approximation (GGA) was employed with the Perdew–Burke–Ernzerhof (PBE) functional. Its widespread success in predicting the structural, mechanical, and hydrogen storage properties of complex hydrides and perovskites supports this choice. GGA-PBE has been shown to underestimate electronic band gaps; however, it is capable of accurately predicting lattice parameters, elastic constants, and formation energies, making it ideal for comparative evaluation of perovskites.^21–25^ We employ the GGA-PBE method in this study to ensure consistency with previous studies and to investigate the effect of A-site substitution on the A_2_LiTiH_6_ system. An ultrasoft pseudopotential was employed that reflects the presence of tightly bound core electrons and characterizes the interaction between electrons and ions. Geometrical optimization yielded a stable three-dimensional electronic structure. A 6 × 6 × 6 Monkhorst–Pack grid was applied to the unit cell in the Brillouin zone for the geometry optimization, which shaped a uniform grid of *k*-points within the space (reciprocal) along all three axes.^26^ For all crystal structures, the cutoff energy of wave functions was set as 600 eV for the expansion of the plane-wave.^27,28^ Through convergence testing, the total energy difference was determined to be <1 meV per atom beyond this cutoff, ensuring accuracy without unnecessary computational cost.^29,30^ The A_2_LiTiH_6_ (X = K, Ca) perovskite-type hydrides formed in a cubical structural frame belonging to the *Fm*-3*m* (no. 225) space group, with lattice angles *α* = *β* = *γ* = 90°. The BFGS minimization algorithm was employed, and structural relaxation was conducted until the energy change was <2 × 10^5^ eV per atom, force < 0.05 eV Å^−1^, stress < 0.1 GPa, and the atoms were allowed to displace <0.002 Å.^31^ Here important to mention that, all CASTEP calculations were carried out under default conditions corresponding to 0 K and 0 Pa, which represent standard DFT practice. These reference conditions ensure that adsorption into the TiH_6_ octahedra is evaluated in its intrinsic lattice-stabilized form, without external temperature or pressure effects. By using these criteria, we ensured that the optimized structure corresponded to the configuration with the lowest energy level in the A_2_LiTiH_6_ (A = K, Ca) system. After the geometry optimization process, the same GGA-PBE framework was utilized for further calculations of electronic band structures, TDOS, PDOS, and optical absorption spectra. To ensure smooth spectral features, a Gaussian smearing of 0.05 eV was applied to the DOS calculations. Based on the frequency-dependent dielectric function, Kramers–Kronig relationships were employed to derive the optical properties, including absorption, conductivity, reflectivity, and refractive index. Since exchange-correlation effects are approximated in GGA-PBE, DFT with GGA-PBE has inherent limitations, particularly the tendency to underestimate band gaps and electronic excitation energies. There is a possibility that these limitations will cause slight shifts in the predicted absorption edges and electronic band positions compared to the experimental values. It has been shown that GGA-PBE can reliably detect qualitative trends in band structure, density of states, and optical behavior in hydride perovskites,^16,21,22,32^ which provides a robust basis for the comparative evaluation of A_2_LiTiH_6_ (X = K, Ca) compounds in this study.

## 3 Results and discussion

### 3.1 Structural properties

**Fig. 1:**
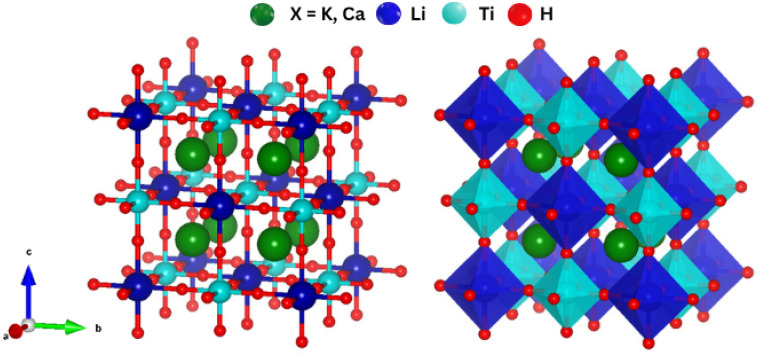
Crystal structure of A_2_LiTiH_6_ (X = K, Ca) perovskite hydrides.

To evaluate geometrical stability and crystallographic characteristics of K_2_LiTiH_6_ and Ca_2_LiTiH_6_ compounds, the GGA-PBE scheme was applied by adopting the same calculation parameters as described in the methodology section. Both compounds adopt cubic symmetry (*a* = *b* = *c*), typical of the double perovskite framework. The space groups are suspected to be similar to those in the *Fm*3*m* prototype, depicted in Fig. 1. In a single crystal unit, constituent atoms were positioned on the Wyckoff positions as follows: Li-atoms →4b(0.5, 0, 0), Ti-atoms were at →4a(0, 0, 0), and H-atoms were at the face center position →24e(0.2513, 0, 0), (K/Ca)-atoms were situated at the corners of the unit cell with coordinates of →8c(0.25, 0.25, 0.75). Hydrogen atoms occupy face-centered positions around the Ti atoms in the A_2_LiTiH_6_ structure, forming TiH_6_ octahedra. The arrangement indicates that hydrogen is structurally integrated into the lattice rather than physiosorbed onto the surfaces. The geometry optimization was achieved using the GGA-PBE exchange-correlation functional, resulting in lattice constants of 8.07 Å for K_2_LiTiH_6_ and 7.54 Å for Ca_2_LiTiH_6_ upon full relaxation. The results correspond to unit cell volumes of 525.39 Å^3^ and 429.17 Å^3^, respectively, as presented in Table 1. In Ca_2_LiTiH_6,_ the reduced lattice constant and cell volume can be ascribed to the smaller ionic radius of Ca^2+^ compared to K^+^, resulting in a tighter and more compact crystal structure.

**Table 1:** Computed structural parameters, tolerance factor (*τ*_G_), formation energy, and cohesive energy for A_2_LiTiH_6_ (A = K, Ca) perovskite hydrides

Compound	Lattice constant (*a* = *b* = *c*) (Å)	Volume (Å)^3^	*τ*_G_	*μ*	Δ*H*_f_(eV per atom)	Δ*H*_f_(kJ mol^−1^ H_2_)	*E*_coh_ (eV per atom)
K_2_LiTiH_6_	8.07	525.39	0.997	0.464	−1.182	−56.96	+1.182
Ca_2_LiTiH_6_	7.54	429.17	0.903	0.464	−1.037	−49.98	+1.037

Perovskite-type materials are frequently predicted by employing the tolerance factor (eqn (1)), which is a dimensionless parameter. The geometric fit of the crystal lattice is determined by the A and B site cations' ionic radii and the anionic radius. When the tolerance factor is close to 1, it indicates that a cubic perovskite structure has formed; a significant deviation from unity, on the other hand, indicates structural distortions or alternative phases. In general, cubic perovskites are stable when their tolerance factor lies between 0.8 and 1.11.^33^1
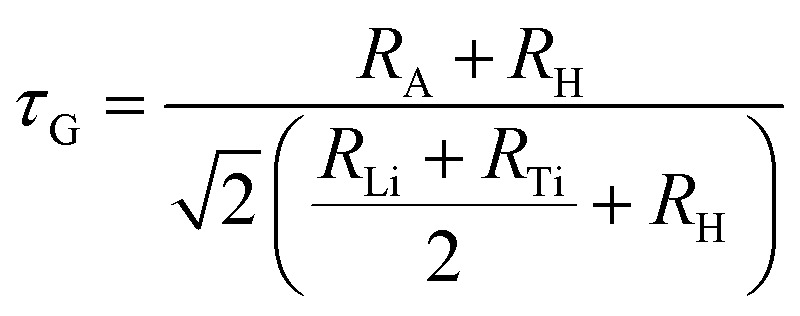


The radius *R*_A_ denotes the ionic radius of the cation at the A-site, the radius *R*_Li_ the ionic radius of the cation at the B-site, the radius *R*_Ti_ the ionic radius of the cation at the B′-site, and the radius *R*_H_ the ionic radius of the anion. Both K_2_LiTiH_6_ and Ca_2_LiTiH_6_ fall within the stability window for the formation of perovskites, as indicated by Goldschmidt tolerance factor values of 0.997 and 0.903, respectively. Ca_2_LiTiH_6_, however, has a slightly more distorted structure due to its lower *τ*_G_ value. Octahedra tilting occurs when octahedral units are deformed angularly within a crystal structure, which is often observed in perovskites. It is caused by the rotation of adjacent octahedra to each other, resulting in structural deformations that significantly affect the material's phase stability and other physical characteristics. When tailoring compounds with specific functionalities, understanding octahedral tilting (eqn (2)) is essential. It is generally accepted that cubic perovskites have an octahedral tilt of between 0.29 and 0.55.^34^2
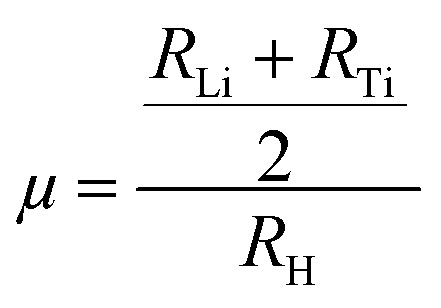
Here, radius *R*_Li_ is the ionic radius of the cation at the B-site, the radius *R*_Ti_ is the ionic radius of the cation at the B′-site, and the radius *R*_H_ is the ionic radius of the anion. Both materials exhibited the same octahedral factor of 0.464, which demonstrates the geometric viability of octahedra with Ti centers in (TiH_6_). Using eqn (3), formation enthalpies for each compound were calculated.^35^3
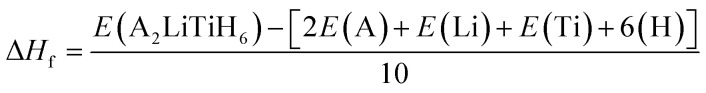
where *E*(A_2_LiTiH_6_) is compositional total energy, *E*(Ae), *E*(Li), *E*(Ti), and *E*(H) are compounds' constituents ground state energies. K_2_LiTiH_6_ had a Δ*H*_f_ of −1.182 eV per atom (−56.96 kJ mol^−1^ H_2_), and Ca_2_LiTiH_6_ had a formation energy of −1.037 eV per atom (−49.98 kJ mol^−1^ H_2_) as represented in Table 1. These results indicate thermodynamic favorability and suggest the possibility of synthesizing the compound under suitable experimental conditions. In K_2_LiTiH_6_, the more negative value is indicative of a slightly higher intrinsic stability than in Ca_2_LiTiH_6_. Furthermore, the cohesive energies, which are a measure of how strongly atoms are bound, were calculated using eqn (4).^36^4
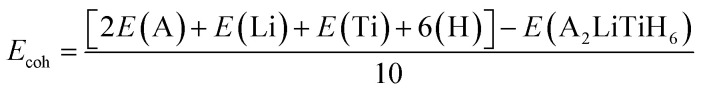
where *E*(Ae), *E*(Li), *E*(Ti), and *E*(H) are compounds' constituents ground state energies and *E*(A_2_LiTiH_6_) is the compound's total energy. The calculated values for the cohesive energies were +1.182 eV per atom and +1.037 eV per atom for K- and Ca-based compounds, respectively, indicating strong bonds within the lattice. As a result of these findings, both K_2_LiTiH_6_ and Ca_2_LiTiH_6_ are structurally and thermodynamically stable candidates, with K substitution resulting in a denser lattice and slightly improved bonding characteristics.

In addition to lattice constants and formation energies, the bond lengths of A_2_LiTiH_6_ compounds provide information regarding chemical bonding and hydrogen storage potential. K_2_LiTiH_6_ and Ca_2_LiTiH_6_ showed the same Ti–H bond lengths of approximately 1.88 Å, which indicates strong covalent interactions between Ti and H atoms. The relatively short Ti–H bonds suggest that hydrogen will bind strongly, which is vital to the stability of the hydride framework. However, *A*–*H* distances were found to be longer, approximately 2.84 Å for K–H and 2.66 Å for Ca–H, which implies weaker interactions. Consequently, hydrogen atoms coordinated near the A-site experience less binding strength, thereby facilitating their potential release during desorption. Due to the presence of strong Ti–H bonds that ensure framework stability and weaker *A*–*H* interactions that enable tunable hydrogen release, this dual bonding behavior highlights the importance of bond length in determining hydrogen storage performance. Asymmetry in bond length has been shown in prior reports on perovskite hydrides to be a strong influence on hydrogen uptake and release.^27,37,38^

### 3.2 Hydrogen storage properties

Recently, hydrogen has appeared as a leading candidate in the transition towards environmentally friendly and clean energy, offering high energy density by weight and zero-carbon emissions at the point of use. However, one of the primary obstacles to its widespread adoption remains the development of a safe, compact, efficient, and suitable storage mechanism. Among the various strategies explored (including liquification and compression), solid-state hydrogen storage (*via* physisorption or chemisorption) is considered an appealing solution. Metal hydrides offer a safer alternative to gaseous or liquid hydrogen, enabling dense hydrogen storage under moderate pressure and temperature conditions.^39^ Particularly, complex hydride perovskites, such as A_2_LiTiH_6_ (A = K, Ca), can be considered for their potential to combine structural stability with favorable thermodynamic behavior. The hydrogen in A_2_LiTiH_6_ is stored as part of TiH_6_ octahedra within the perovskite lattice, and its desorption occurs through a reversible release mechanism, which distinguishes this process from surface adsorption. In evaluating the hydrogen storing potential of a compound, a key parameter, *i.e.*, gravimetric capacity (*C*_ωt%_), refers to the mass percentage of hydrogen relative to the total mass of the storage compound. This metric is critical for mobile applications where weight efficiency is paramount. In this study, the calculated *C*_ωt%_ for K_2_LiTiH_6_ and Ca_2_LiTiH_6_ were 4.38 and 4.29 wt%, respectively, calculated using eqn (5).^40^5
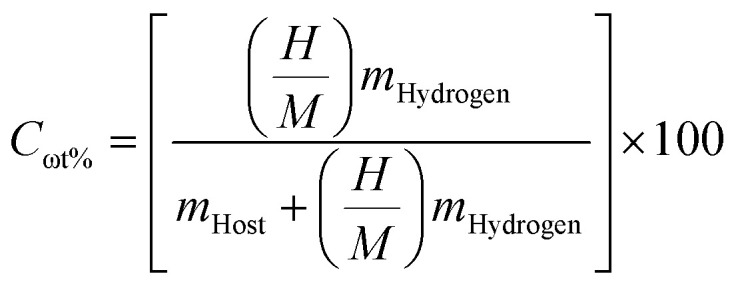
Here, *m*_Hydrogen_ and *m*_Host_ denote the molar masses of hydrogen, and hosting complex composition, respectively, and 
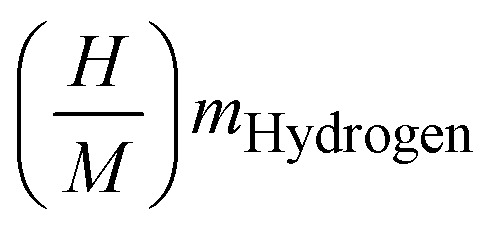
 represents the ratio of hydrogen atoms to host-complex composition atoms. Slightly higher hydrogen content in K_2_LiTiH_6_ is attributed to its more favorable host-to-hydrogen mass ratio (molar mass of K < molar mass of Ca), making it a marginally better candidate for high-capacity storage. Notably, while both materials show promising characteristics, their gravimetric capacities remain just below the 2025 U.S. Department of Energy target of 5.5 wt%. It is noteworthy that our compounds display highly competitive gravimetric capacities compared to other double perovskites. As an example, Azeem *et al.*^14^ reported a gravimetric capacity of 1.39 wt% for Cs_2_CaCdH_6_ and 1.69 wt% for Rb_2_CaCdH_6_. Obeidat *et al.*^41^ reported gravimetric capacities for K_2_NaXH_6_ (X = Al, As, Bi Ga, In) that range from 1.913 to 4.506 wt%, with K_2_NaAlH_6_ exhibiting the most incredible capacity for hydrogen storage. Furthermore, Ahmad *et al.*^16^ found 3.86 wt% and 2.40 wt% for Ca_2_LiCuH_6_ and Sr_2_LiCuH_6_, respectively, which are lower than the values found in the current study for both K_2_LiTiH_6_ and Ca_2_LiTiH_6_.

Another parameter for evaluating the potential in the field of hydrogen storage applications of a compound is volumetric hydrogen capacity (*ρ*_vol_). This parameter is defined as the amount of hydrogen that can be stored per unit volume of a material, which is especially important for space-limited applications. It is calculated using hydrogen no. of atoms, the hydrogen molar mass, the material's unit cell volume, and Avogadro's number by eqn (6).^42^6
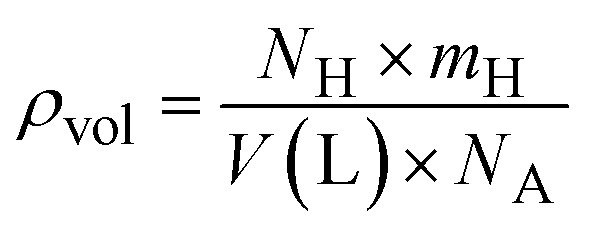


Herein, *N*_H_, *m*_H_, *V*(L) and *N*_A_ represent absorbed hydrogen amount (*i.e.*, no. of hydrogen atoms in a unit cell), hydrogen's molecular weight, absorbent's volume (volume of unit host lattice cell) and the Avogadro number, respectively. For A_2_LiTiH_6_ (A = K, Ca) compounds, the measured volumetric capacities (hydrogen storing) are 19.12 g L^−1^ for K_2_LiTiH_6_ and 23.41 g L^−1^ for Ca_2_LiTiH_6_. The trend corresponds to increasing unit cell volume across the series. Among these, Be_2_LiAlH_6_ offers the highest volumetric capacity, which is closest to the U.S. DOE 2025 target of 40 g L^−1^, highlighting its suitability for compact hydrogen storage systems.

Beyond storage capacity, hydrogen release behavior, which is quantified by the hydrogen desorption temperature (*T*_des_) is equally important. For practical use, especially in conjunction with proton exchange membrane fuel cells (PEMFCs), hydrogen must be released within a moderate temperature window (typically between 233 and 333 K). It is important to note that in this study hydrogen storage refers to chemisorption within the TiH_6_ octahedra under standard DFT conditions (0 K, 0 Pa), whereas the subsequent desorption temperatures are thermodynamically derived from formation enthalpies and entropy considerations. The thermodynamics of the material fundamentally governs desorption temperature and can be estimated using the Gibbs free energy, measured by the eqn (7).

7 Δ*G* = Δ*H* − *T*_des_ × Δ*S*

In this equation, Δ*G* represents the Gibbs free energy change; Δ*H* represents the enthalpy change due to hydrogen desorption; Δ*S* represents the entropy change; and *T*_des_ represents the desorption temperature. This approach relates the desorption temperature to the material's enthalpy and entropy of formation. In standard equilibrium conditions (∼300 K, ∼1 atm), the temperature at which the Gibbs free energy change becomes zero corresponds to the point where hydrogen desorption occurs. *T*_des_ can be estimated by the eqn (8):^43^8
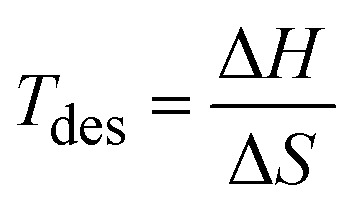
where *T*_des_ is the hydrogen desorption temperature, Δ*H* is the enthalpy of formation per H_2_ molecule, and Δ*S* is the entropy change. Assuming a standard entropy change for hydrogen gas (approximately 130.7 J mol^−1^ K^−1^), the measured desorption temperatures (435.8 K for K_2_LiTiH_6_ and 380.4 K for Ca_2_LiTiH_6_) were found to exceed the ideal operational range for onboard fuel cell applications. These results are comparable to those reported for double perovskite hydrides.^14,16,41^ Nevertheless, the relatively higher desorption temperatures do not preclude the use of these compounds in field hydrogen storage. On the contrary, they remain highly viable for off-board storage systems, where hydrogen can be released under controlled heating conditions. Among the compounds, Ca_2_LiTiH_6_ again stands out with the most favorable combination of high storage capacity and relatively lower desorption temperature compared to K_2_LiAlH_6_. The calculated gravimetric ratios, volumetric hydrogen capacities and desorption temperatures are listed in Table 2. This suggests that tailored chemical substitutions such as partial doping or alloying could further optimize the balance between capacity and thermodynamics. These findings highlight Ca_2_LiTiH_6_ as the most promising candidate for implications in the field of hydrogen storing, offering the best combination of high gravimetric and volumetric capacities along with favorable thermodynamic behavior, making it well-suited for off-board storage and a strong contender for future hydrogen energy systems. Ca_2_LiTiH_6_ exhibits a relatively higher hydrogen storage capacity and a lower desorption temperature due to its smaller ionic radius as compared to K^+^. Consequently, the lattice constant is reduced, atomic packing is denser, and Ti–H bond lengths are shorter, all of which enhance hydrogen binding and facilitate moderate-temperature desorption. To further highlight the relevance of computed results, we compared our findings with previously reported double perovskite hydrides (Table 2). The *C*_ωt%_ and *T*_des_ of studied compositions are better than other double perovskite hydrides. This comparison demonstrates the advantage of incorporating lightweight cations (Li, Ti, Ca, K) in achieving a favorable balance between hydrogen capacity and thermodynamic stability, thereby establishing the necessity and superiority of this study over prior work.

**Table 2:** The volumetric hydrogen capacities, desorption temperatures and gravimetric ratios of A_2_LiTiH_6_ (X= K, Ca) perovskite hydrides

Compound	*T*_des_ (K)	*ρ*_vol_ (g H_2_ L^−1^)	*C*_wt_ (%)	Ref.
Rb_2_AlInH_6_	113.75	15.496	1.46	44
Rb_2_AlTlH_6_	120.98	15.283	1.86	44
Ca_2_LiCuH_6_	717.2	15.68	3.86	16
K_2_LiInH_6_	—	—	2.90	45
K_2_NaAlH_6_	484.52	74.25	4.47	42
Rb_2_AsSnH_6_	—	—	1.63	46
Cs_2_NaInH_6_	492.7	14.18	1.48	47
Cs_2_AlInH_6_	534.15	14.18	1.20	47
K_2_LiTiH_6_	435.8	19.12	4.38	Present
Ca_2_LiTiH_6_	380.4	23.41	4.29	Present

### 3.3. Mechanical properties

A material's mechanical behavior is an essential consideration when determining whether it is suitable for practical applications, particularly in hydrogen storage systems that are subject to pressure and cyclic loading.^48^ As hydrogen storage is directly dependent on these mechanical characteristics, stable elastic moduli are necessary to maintain structural integrity during repeated hydrogen absorption and desorption cycles. In this study, we calculated second-order elastic constants (*C*_*ij*_) for A_2_LiTiH_6_ perovskite hydrides (X = K, Ca), and derived key mechanical parameters to evaluate their mechanical properties. Besides revealing the fundamental mechanical stability of these hydrides, these also shed light on their ductility, stiffness, anisotropy, and bonding nature, all of which have bearing on their performance in hydrogen storage environments. As a result of different modes of stress application, these constants reflect the material's response: the *C*_11_ values are indicative of the longitudinal elastic response along the principal axes and indicate resistance to axial compression or tension. To assess mechanical stability under multi-axial loads, the coupling between everyday stresses and shear strains is crucial, as *C*_12_ represents the interaction between everyday stresses and shear strains. *C*_44_ is the material's resistance to shear deformation in planes perpendicular to its principal axes. In Table 3, the elastic constants *C*_11_, *C*_12_, and *C*_44_ are summarized.^49^ According to the Born stability criteria^50^ (*C*_11_ > 0; *C*_44_ > 0; *C*_11_ + 2*C*_12_ > 0), cubic systems A_2_LiTiH_6_ meet the requirements.

**Table 3:** The computed elastic constants (*C*_*ij*_) and born stability criterion; for A_2_LiTiH_6_ (A = K, Ca) perovskite hydrides

Compounds	Elastic constant (*C*_*ij*_)						Stability
	*C*_11_	*C*_12_	*C*_44_	*C*_11_ > 0	*C*_44_ > 0	*C*_11_ + 2*C*_12_ > 0	
K_2_LiTiH_6_	43.68	18.12	28.18	43.68	28.18	79.92	Stable
Ca_2_LiTiH_6_	108.83	23.40	34.92	108.83	34.92	155.63	Stable

Bulk modulus (*B*) describes the ability of a compound to resist compression (uniformly), which is necessary in hydrogen storage systems to withstand external pressures. When it comes to materials that are expected to retain their structural integrity, higher *B* values are preferable. Measuring the rigidity of a material is the ability to withstand shape change without volume change. Hydrogen absorption/desorption may cause shear stresses, so higher shear modulus (*G*), values are advantageous for maintaining mechanical integrity. Young modulus (*E*) is a quantity of stiffness along the principal loading direction. It is critical in evaluating deformation as a result of mechanical loading. In accordance with Voigt–Reuss–Hill formulism for polycrystalline compounds the elastic moduli were calculated.^51^Table 4 provide comparison of elastic parameters *B*, *G*, and *E*. These parameters of K_2_LiTiH_6_ are 26.64 GPa, 20.52 GPa, and 48.98 GPa, respectively. This indicates a relatively low degree of resistance to volume and shape changes. On the other hand, Ca_2_LiTiH_6_ shows significantly higher moduli (*B* = 51.88 GPa, *G* = 37.85 GPa, and *E* = 91.34 GPa), which indicates enhanced rigidity and stiffness in comparison with potassium.

**Table 4:** Elastic constants calculated for A_2_LiTiH_6_ (X = K, Ca) perovskite hydrides

Compounds	*B*	*G*	*E*	*B*/*G*	*ν*	*C*′	*A*_U_
K_2_LiTiH_6_	26.64	20.52	48.98	1.29	0.19	12.78	0.79
Ca_2_LiTiH_6_	51.88	37.85	91.34	1.37	0.21	42.72	0.05

The bulk-to-shear modulus ratio, also acknowledged as Pugh's ratio, provides information about the ductility of materials. *B*/*G* > 1.75 indicate ductility, while values <1.75 indicate brittilitty.^52^ K_2_LiTiH_6_ and Ca_2_LiTiH_6_ have *B*/*G* ratios of 1.29 and 1.37, respectively. Although these values are below the threshold of ductility, they suggest a mixed brittle-ductile character, with Ca_2_LiTiH_6_ having a slightly higher ductility. Further support for these findings comes from Poisson's ratios^53^ of 0.19 for K_2_LiTiH_6_ and 0.21 for Ca_2_LiTiH_6_, which indicate that both materials are moderately brittle. The anisotropy factor (*A*_U_), which measures elastic anisotropy,^54^ is lower for Ca_2_LiTiH_6_ (0.05) than for K_2_LiTiH_6_ (0.79), suggesting that the calcium-containing compound has a more isotropic elastic property. Those with negative values are characterized by directional bonding and are prone to covalent bonding, which corresponds with their mixed brittle-ductile nature.^55^ It can be concluded that the mechanical characteristics of K_2_LiTiH_6_ and Ca_2_LiTiH_6_, namely the bulk modulus, shear modulus, elastic constants, and Poisson's ratio, play an important role in determining the suitability of these materials for hydrogen storage applications. As a result of the high bulk modulus of these compounds (especially Ca_2_LiTiH_6_), these compounds are capable of withstanding high pressures without experiencing significant deformation, which is essential for the storage of hydrogen under pressure. Moreover, both materials exhibit sufficient structural integrity to withstand the cyclic loading and unloading experienced during hydrogen absorption and desorption, as indicated by their shear modulus and Poisson's ratio. Together, these mechanical characteristics highlight the stability, durability, and reliability of K_2_LiTiH_6_ and Ca_2_LiTiH_6_ for use as hydrogen storage systems, ensuring their performance over extended cycles and under practical storage conditions.

### 3.4. Electronic properties

Hydrogen storage materials need to be examined in terms of their electronic structure since it directly impacts adsorption–desorption behavior, chemical stability, and potential catalytic activity toward hydrogen dissociation or recombination.^56^ As part of our Investigation into the fundamental electronic behavior and its implications in the field of storing hydrogen, we conducted an extensive analysis of the electronic band structure, total density of states (TDOS), and partial density of states (PDOS) for both compositions. GGA-PBE was used to perform these calculations.

**Fig. 2:**
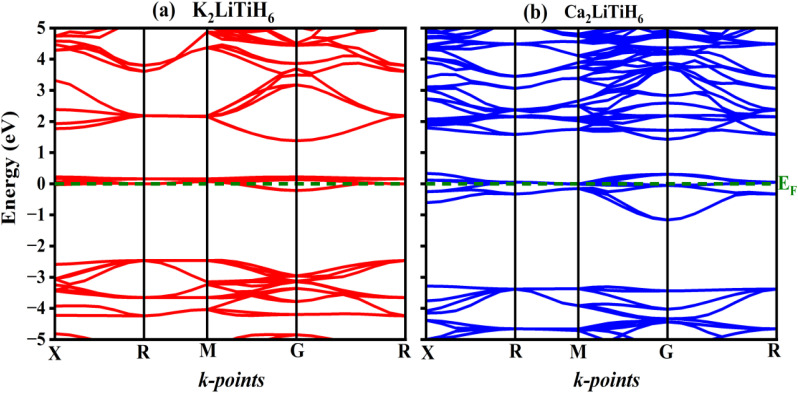
Band structure for the (a) K_2_LiTiH_6_ and (b) K_2_LiTiH_6_ hydrides.

An analysis of a material's band structure can provide invaluable insights into its electronic stability, conductivity, and suitability for energy-related applications.^57^ In hydrogen storage materials, the band structure affects electron mobility and availability, directly impacting hydrogen binding, release kinetics, and overall thermodynamic stability. The electronic band structures of K_2_LiTiH_6_ and Ca_2_LiTiH_6_ are depicted in Fig. 2(a and b). Upon closer examination of their electronic band structures, it is revealed that there is a subtle^58^ but significant overlap near the Fermi level. As a result of this partial overlap, weak metallic or semi metallic tendencies are apparent, which may not be prominent enough to be classified as true metals but can have an effect on their electronic conductivity and hydrogen desorption properties. Hydrogen storage materials may benefit from near-Fermi-level overlap in two ways. Aside from facilitating charge transfer, it may aid hydrogen molecule dissociation and improve desorption kinetics. Conversely, excessive metallic behavior can compromise the reversibility and stability of hydrogen uptake through the formation of unintended electronic excitations or distortions of the lattice due to hydrogen.^59^

**Fig. 3:**
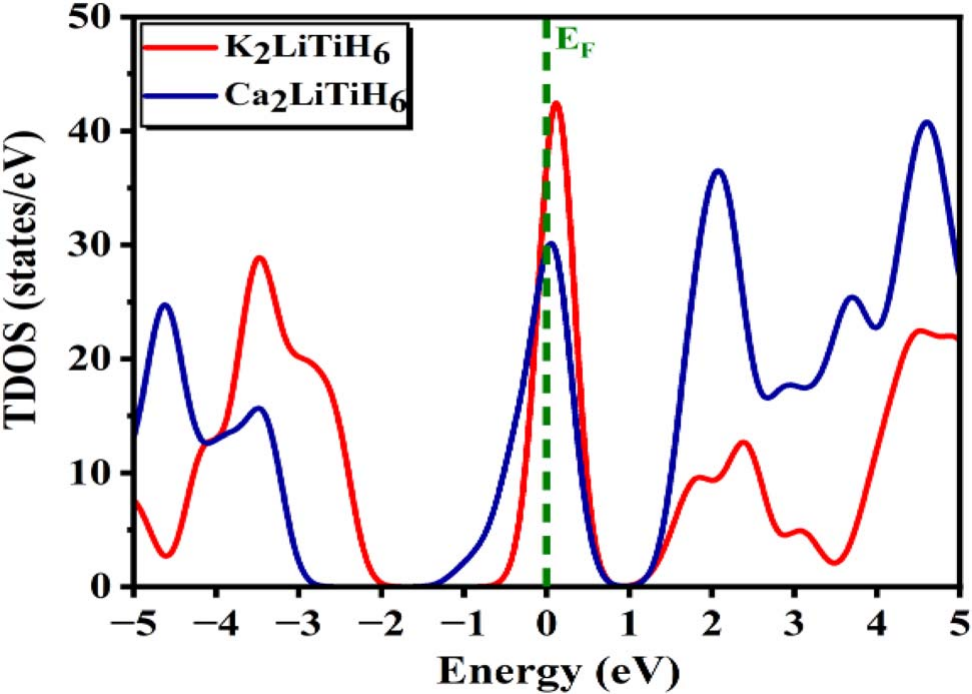
Total density of states for the A_2_LiTiH_6_ (A = K, Ca) hydrides.

**Fig. 4:**
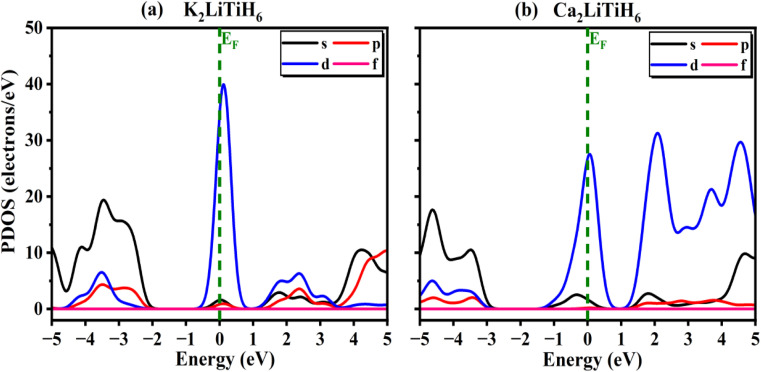
PDOS for the (a) K_2_LiTiH_6_ and (b) K_2_LiTiH_6_ hydrides.

Based on the TDOS and PDOS plots in Fig. 3 and 4, one can get more understanding into the electronic behavior near the Fermi level and understand how orbital contributions play a role in the observed band behaviour. According to the TDOS plots (Fig. 3), the band structure findings for both compositions are corroborated by the TDOS and PDOS plots. The regions in both valence band and conduction band of compositions is primarily composed of strongly hybridized s, p and d states. In K_2_LiTiH_6_ compared to Ca_2_LiTiH_6_, the density of electronic states near the valence band maximum (VBM) is significantly higher, suggesting greater availability of occupied electronic states for hole transport. In contrast, in the conduction band region, Ca_2_LiTiH_6_ has a higher density of unoccupied states near the conduction band minimum (CBM), which may facilitate electron intensification. The differences between the DOS profiles of K_2_LiTiH_6_ and Ca_2_LiTiH_6_ are primarily the result of orbital contributions near the Fermi level. Ca_2_LiTiH_6_ exhibits better desorption kinetics due to an increased availability of unoccupied Ti-3d states in the conduction band minimum, which allows hydrogen to be dissociated and desorption kinetics to be improved.

A subtle overlap is observed near the Fermi level in both K2LiTiH6 and Ca2LiTiH6, suggesting a weakly metallic or semi-metallic nature. It is believed that this property reflects the presence of partially delocalized electronic states that enhance electrical conductivity.^60^ It is crucial for hydrogen adsorption and desorption dynamics to have a metallic-like conductivity, since this facilitates charge transfer processes. Particularly, partial filling of conduction states may reduce the energy barrier for hydrogen molecule dissociation, improving desorption kinetics. Furthermore, excessive overlap could compromise reversibility by destabilizing the hydride lattice; the modest overlap observed here indicates a balanced effect, which maintains structural stability while enabling favorable charge transport. Due to this dual behavior, the Fermi-level electronic structure plays a vital role in tuning the hydrogen storage properties of perovskites.

### 3.5. Dynamic stability

**Fig. 5:**
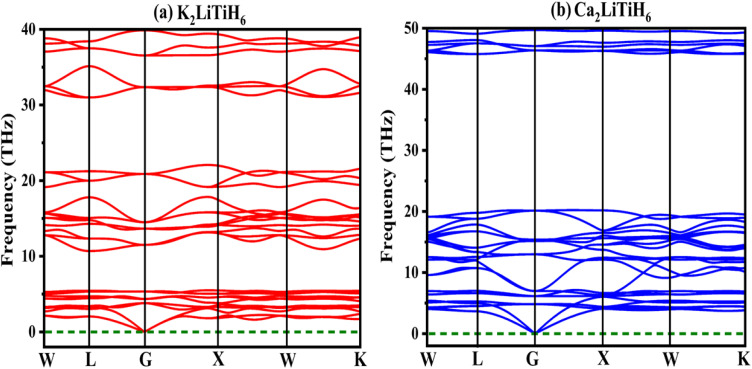
Phonon dispersions describing (a) K_2_LiTiH_6_ and (b) K_2_LiTiH_6_ hydrides.

For assessing dynamical stability and understanding the lattice dynamics of materials, which have significant effects on their thermal, mechanical, and transport properties, a phonon dispersion analysis is essential.^61^ Dynamic stability is critical for the real-world application of hydrogen storage materials as they enable them to undergo multiple cycles without losing efficiency or functionality. To verify the dynamical stability of the examined structure, phonon dispersion relations were calculated across the first Brillouin zone.^62,63^ In crystalline solids, phonon band structures are critical indicators of vibrational stability, since imaginary (negative) phonon frequencies indicate dynamic instability and possible structural distortions. To confirm that the optimized geometry corresponds to an actual minimum on the potential energy surface, it is necessary to assess the vibrational spectrum. It can be seen from Fig. 5(a) and (b) that no imaginary modes are observed in the Brillouin zone, confirming the stability of the system.^64^ As expected, the acoustic branches approach zero at the *G*-point smoothly, while the optical branches are well separated, exhibiting distinct vibrational modes at the *G*-point. Soft phonon modes do not appear to occur in the structure, suggesting that the optimized configuration is not subject to lattice distortions or symmetry-breaking displacements, indicating that the optimized configuration is in fact a minimum-energy state rather than a saddle point.^65^

Furthermore, the vibrational spectrum indicates that the lattice vibrations are well confined, and no anomalous softening occurs along the high-symmetry directions. The results are in agreement with those derived from elastic constant analysis (see Section 3.3), demonstrating the robustness of the investigated compound. This study confirms the dynamic stability of the studied material under ambient conditions. Although the present analysis is limited to zero-temperature phonon calculations, it provides a solid basis for determining the thermodynamic stability of the system.

### 3.6. Optical properties

To integrate materials into optoelectronic and energy-related applications, it is considered essential to understand the optical performance of the compounds.^66^ A comprehensive investigation of the optical characteristics of A_2_LiTiH_6_ (X = K, Ca) was performed by using DFT within the GGA-PBE approximation in this study. The analysis encompasses the complex dielectric function, optical conductivity, and energy loss spectrum, providing a comprehensive overview of light–matter interactions in the materials.

**Fig. 6:**
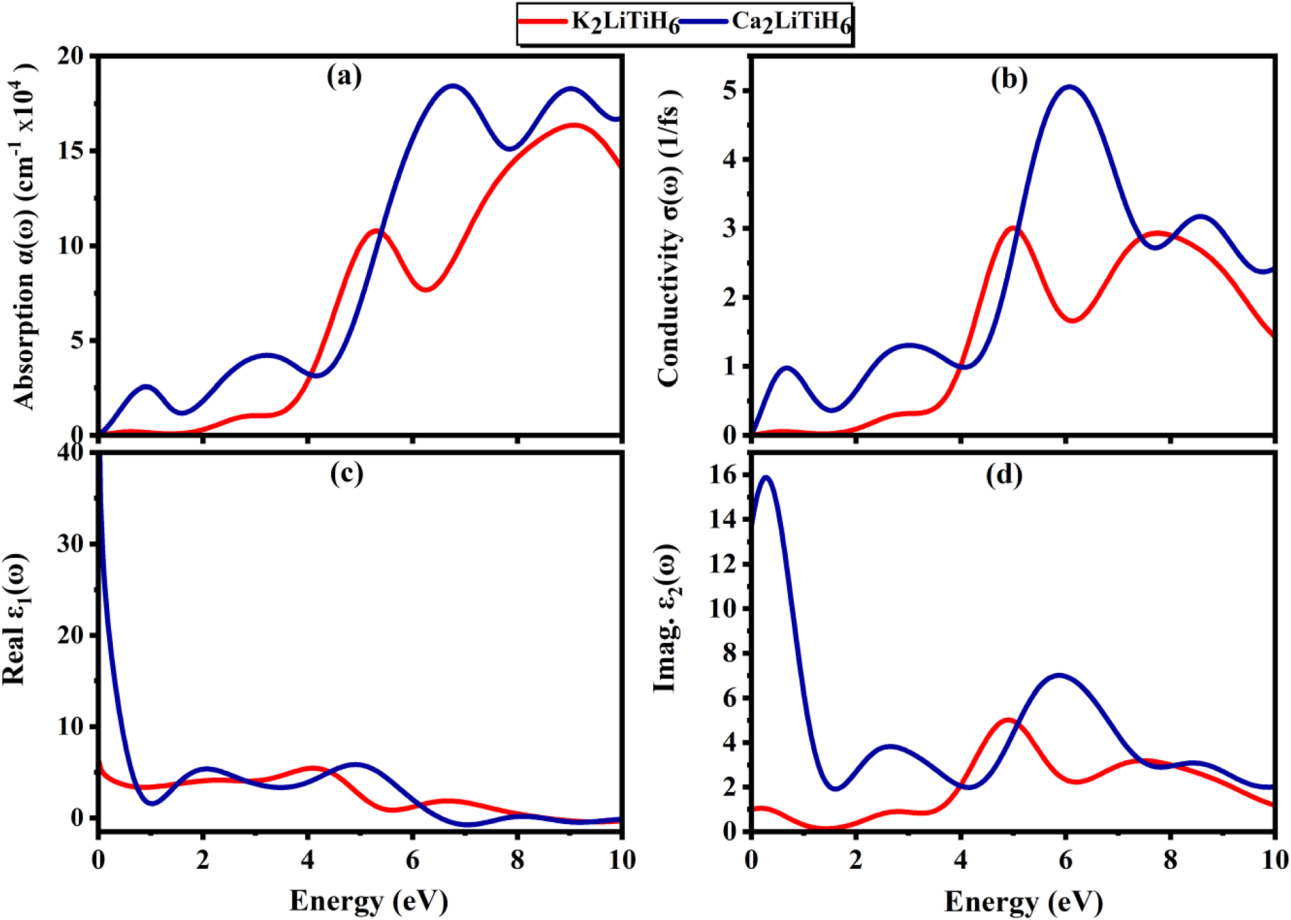
(a) Absorption and (b) conductivity, (c) real part of dielectric function, (d) imaginary part of dielectric function.

The dielectric function is crucial for comprehending and creating optical and electrical devices in materials science.^67^ According to the Ehrenreich–Cohen formalism,^68^ the optically performance of the material is assessed by the complex dielectric function, *ε*(*ω*) (eqn (9)):

9 *ε*(*ω*) = *ε*_1_(*ω*) + *i*·*ε*_2_(*ω*)

where *ε*_1_(*ω*) is the real part, and *ε*_2_ (*ω*) is the imaginary part of *ε*(*ω*). Dielectric functions (Fig. 6(c)) provide detailed information concerning polarization and photon energy absorption.^69^ There is a significant difference in static dielectric constant between Ca_2_LiTiH_6_ (35) and K_2_LiTiH_6_ (5), indicating that Ca_2_LiTiH_6_ has a much stronger polarizability and screening effect. Ca_2_LiTiH_6_ exhibits steep rises and broad features between 2 and 8 eV, whereas K_2_LiTiH_6_ exhibits a more subdued response. The imaginary component for Ca_2_LiTiH_6_ exhibits increased peaks at lower photon energies (2 eV and 6 eV), illustrating stronger interband transitions and optical absorption. It appears that K2LiTiH6 exhibits weaker optical transitions and lower light absorption, resulting from decreased intensity and delayed peaks.

Fig. 6(c and d) illustrate the *ε*_1_(*ω*) and *ε*_2_(*ω*), which provides insight into the polarization response and energy absorption mechanisms of the materials under electromagnetic stimulation. Based on calculations with zero photon energy, K_2_LiTiH_6_ has a static dielectric constant of 4.99, and Ca_2_LiTiH_6_ has an exceptionally high static dielectric constant of 34.74. This high value for Ca_2_LiTiH_6_ suggests a pronounced polarizability and potentially strong screening effects, probably due to the denser electronic environment and increased levels of free carriers. Ca_2_LiTiH_6_ exhibits a more prominent peak intensity and broader spectral characteristics than K_2_LiTiH_6_, which indicates a greater capacity for photoinduced polarization.

An absorption coefficient is proportional attenuation of light per unit length within a compound, which quantifies how rapid light intensity decreases as a function of thickness.^70^ By analyzing the dielectric function's real and imaginary parts, absorption coefficient (*α*(*ω*)) behavior can be calculated using the following (Eq. (10)):10
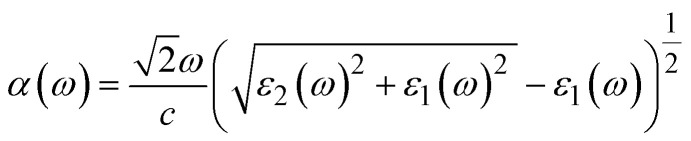
where, *ε*_1_(*ω*) is the real part of the dielectric function, *ε*_2_(*ω*) is the imaginary part of the dielectric function, *ω* is the angular frequency of the photon, and *c* is the speed of light. For both compounds, the absorption spectra shown in Fig. 6(a) demonstrate significant absorption in the UV-visible range. With a peak near 7 eV, Ca_2_LiTiH_6_ exhibits a significantly higher absorption coefficient than K_2_LiTiH_6_. It indicates that calcium-containing compounds have a higher density of electronic states and a greater ability to interact with light. In addition, both materials exhibit negligible absorbance under 1 eV, consistent with their zero-bandgap nature. Based on the optical conductivity spectra presented in Fig. 6(b), it is possible to visualize the dynamic response of free carriers to optical excitation. These materials exhibit broad conductivity characteristics across a wide range of energies, reflecting continuous electronic transitions characteristic of metallic or semi-metallic systems. Ca_2_LiTiH_6_ exhibits a significantly higher conductivity peak, with maxima near 5 eV and 8 eV, reaching a value of up to 5 (fs). Conversely, K_2_LiTiH_6_ exhibits more moderate peaks, around 3 (fs). In accordance with its elevated static dielectric constant, these findings support the enhanced charge carrier dynamics and higher free carrier density in Ca_2_LiTiH_6_. As a result of the metallic/semi-metallic features observed, there is a direct correlation with hydrogen storage: enhanced electronic conductivity facilitates charge transfer, which in turn regulates the dissociation and desorption of hydrogen.

**Fig. 7:**
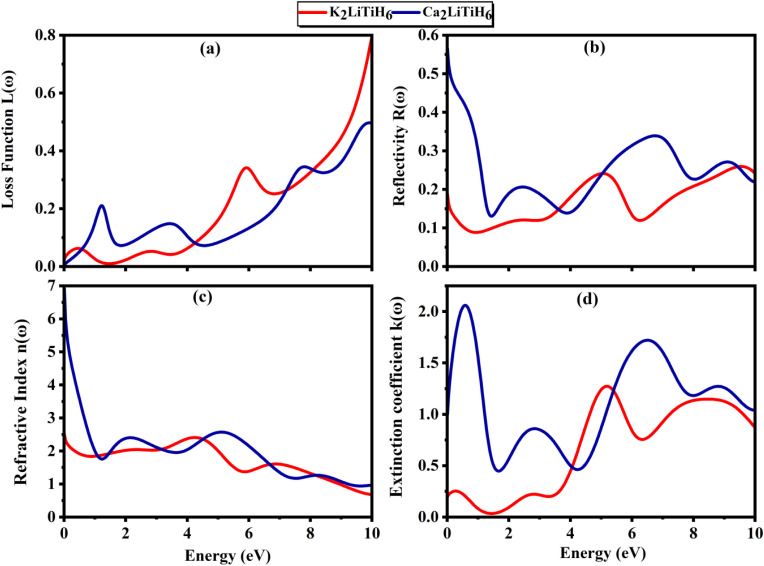
(a) Loss function, (b) reflectivity, (c) refractive index and (d) extinction coefficient.

This energy loss function describes the energy losses by fast electrons as they traverse the material and is related to plasmon resonance, calculated employing eqn (11).11
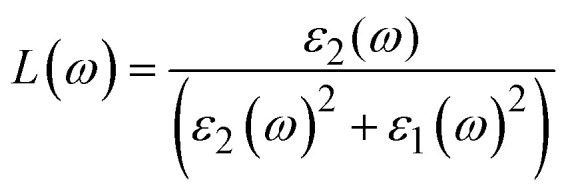
where, *L*(*ω*) presents the energy loss function, *ε*_1_(*ω*) and *ε*_2_(*ω*) are real part and imaginary part of dielectric function, respectively. The energy loss spectra (Fig. 7(a)) provide information regarding plasmon resonances and collective excitations, which are both characterized by inelastic scattering of electrons. As compared to K_2_LiTiH_6_, Ca_2_LiTiH_6_ exhibits a more intense and broader loss function, indicating stronger plasmonic behavior and a higher free-electron density, which is in accordance with its metallic nature and high optical characteristics.

The refractive index, *n*(*ω*), is a vital property which defines the relative velocity with which light propagates within a compound in comparison to its vacuum value. The electromagnetic field interacts with the electrical charges on the material to modify light velocity. By measuring the extinction coefficient of the material, we can find attenuation that causes to the incident electromagnetic wave due to absorption. Dielectric functions related to optical refractive index (*n*) and extinction coefficient (*k*) as (eqn (12a) and (12b)):^71^

12a *ε*_1_(*ω*) = *n*^2^ − *K*^2^

12b *ε*_2_(*ω*) = 2*nK*

In Fig. 7(c) the refractive index *n* trends closely follow the behavior of the dielectric medium, with static values consistent with *ε*(0). Ca_2_LiTiH_6_ has a higher refractive index, corresponding to its enhanced dielectric response, indicating that it is capable of bending light more readily and has the potential for high-index optical devices.

Extinction coefficient profiles (Fig. 7(d)) illustrate the attenuation of incident light caused by absorption and scattering mechanisms. Compared with K_2_LiTiH_6_, Ca_2_LiTiH_6_ exhibits significantly broader and more intense peaks, suggesting its superior ability to dissipate photon energy, making it useful for optical filtering or shielding applications. This behavior is consistent with the material's higher optical conductivity and *ε*_2_(*ω*). Further confirmation of these trends can be found in Fig. 7(b). K_2_LiTiH_6_ shows a static reflectivity (R(0)) of 0.16, while Ca_2_LiTiH_6_ has an elevated reflection (*R*(0)) of 0.52, indicating that a substantial portion of incident light is reflected at the surface. Both compounds exhibit increasing reflectivity with increasing photon energy, with Ca_2_LiTiH_6_ showing a greater reflectance across the entire spectral range.

### 3.7. Thermodynamical properties

The thermal and acoustic properties of K_2_LiTiH_6_ and Ca_2_LiTiH_6_ double perovskite hydrides were analytically inspected to evaluate their thermodynamic stability and potential for optoelectronic applications. The Debye temperature (*θ*_D_), one of the fundamental parameters that govern lattice vibrations and thermal conductivity, is calculated using eqn (13):^72^13
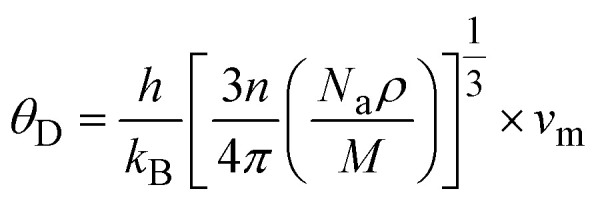


This equation has four constants: Na is Avogadro's number, *h* is Planck's constant, *k*_B_ is Boltzmann's constant, *n* is atom number, and *M* is molar mass. As *θ*_D_ equation based on mainly average sound velocity (*v*_m_) which is calculated using eqn (14):^73^14
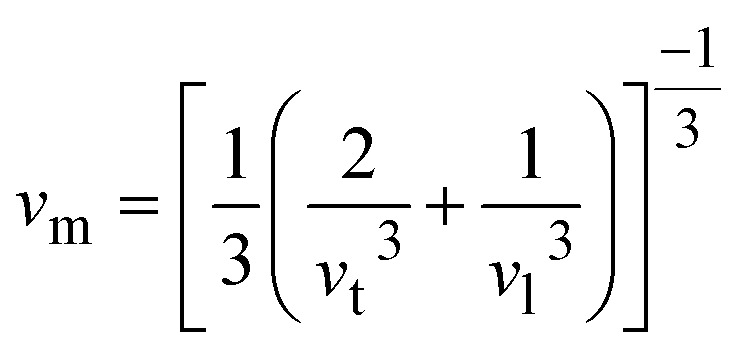
where *v*_l_ and *v*_t_ denote longitudinal and transverse sound velocities, respectively, and calculated using eqn (15):^74^15
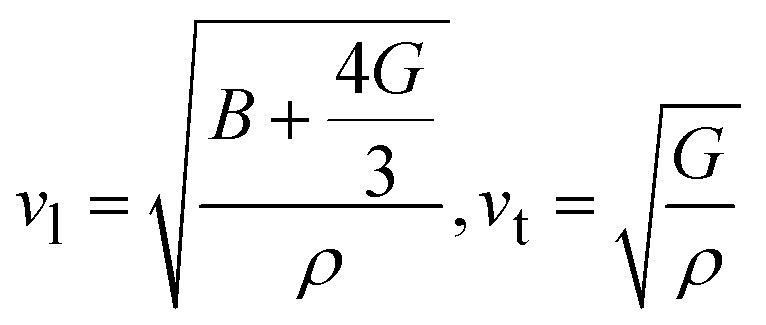
Here, in these terms *G*, *B*, and *ρ* are the shear, bulk modulus density, respectively. According to Table 5, Ca_2_LiTiH_6_ exhibits a significantly higher Debye temperature (*θ*_D_ = 584.0 K) compared to K_2_LiTiH_6_ (*θ*_D_ = 447.2 K), suggesting stronger atomic bonding and superior thermal stability in the Ca-based double perovskite. This enhancement in thermal characteristics is attributed to the greater elastic moduli of Ca_2_LiTiH_6._ with *B* = 51.88 GPa and *G* = 37.85 GPa, in contrast to K_2_LiTiH_6_, which has *B* = 26.64 GPa and *G* = 20.52 GPa. Correspondingly, *v*_l_ and *v*_t_ are also elevated for Ca_2_LiTiH_6_, with *v*_l_ = 3466.4 ms^−1^ and *v*_t_ = 5700.1 ms^−1^, compared to *v*_l_ = 2842.3 ms^−1^ and *v*_t_ = 4610.8 ms^−1^ for K_2_LiTiH_6_. These higher velocities contribute to an increased average sound velocity (*v*_m_ = 3829.6 ms^−1^ for Ca_2_LiTiH_6_*vs.* 3135.7 ms^−1^ for K_2_LiTiH_6_), which directly influences the Debye temperature as per the eqn (16). The observed trend reflects a denser and more rigid atomic structure in Ca_2_LiTiH_6,_ thereby affirming its greater phonon transport efficiency and thermal robustness compared to its potassium-based counterpart.

**Table 5:** Thermal parameters calculated for A_2_LiTiH_6_ (X = K, Ca) perovskite hydrides

Compounds	*v*_l_ (ms^−1^)	*v*_t_ (ms^−1^)	*v*_m_ (ms^−1^)	*T*_m_ (K)	*H*_v_ (GPa)	*θ*_D_ (K)
K_2_LiTiH_6_	2842.3	4610.8	3135.7	811.17	9.64	447.2
Ca_2_LiTiH_6_	3466.4	5700.1	3829.6	1195.16	17.10	584.0

It is necessary to study the advanced materials' melting temperature (*T*_m_) to determine how well they will perform at different temperatures. With the help of this parameter, one can calculate the cubic crystal structure following the elastic constants by the following equation. 16:^75^

16 *T*_m_ = 553 + 5.91*C*_11_

The evaluated melting temperatures, shown in Table 5, confirm the suitability of these double perovskites for thermal applications. Ca_2_LiTiH_6_ demonstrates greater thermal stability with a *T*_m_ of 1195.16 K, while K_2_LiTiH_6_ exhibits a lower *T*_m_ of 811.17 K. This difference suggests that Ca-based perovskites may be more thermally robust during high-temperature fabrication and operation processes.

The Hardness (*H*_v_) for the double perovskite materials is calculated using the eqn (17):^76^17
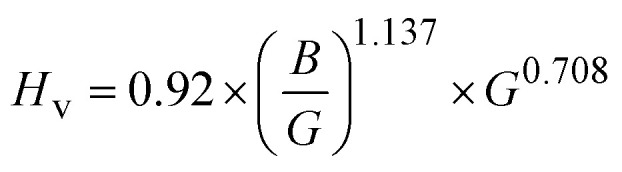


The calculated hardness values (Table 5) show a significant increase from 9.64 GPa for K_2_LiTiH_6_ to 17.10 GPa for Ca_2_LiTiH_6_. This indicates that the Ca-based perovskite not only has superior thermal stability but also retains greater mechanical strength under stress. Due to their high Debye temperatures and melting points, these compounds can withstand thermal cycling during hydrogen release processes, ensuring a high degree of reliability under normal operating conditions.

**Fig. 8:**
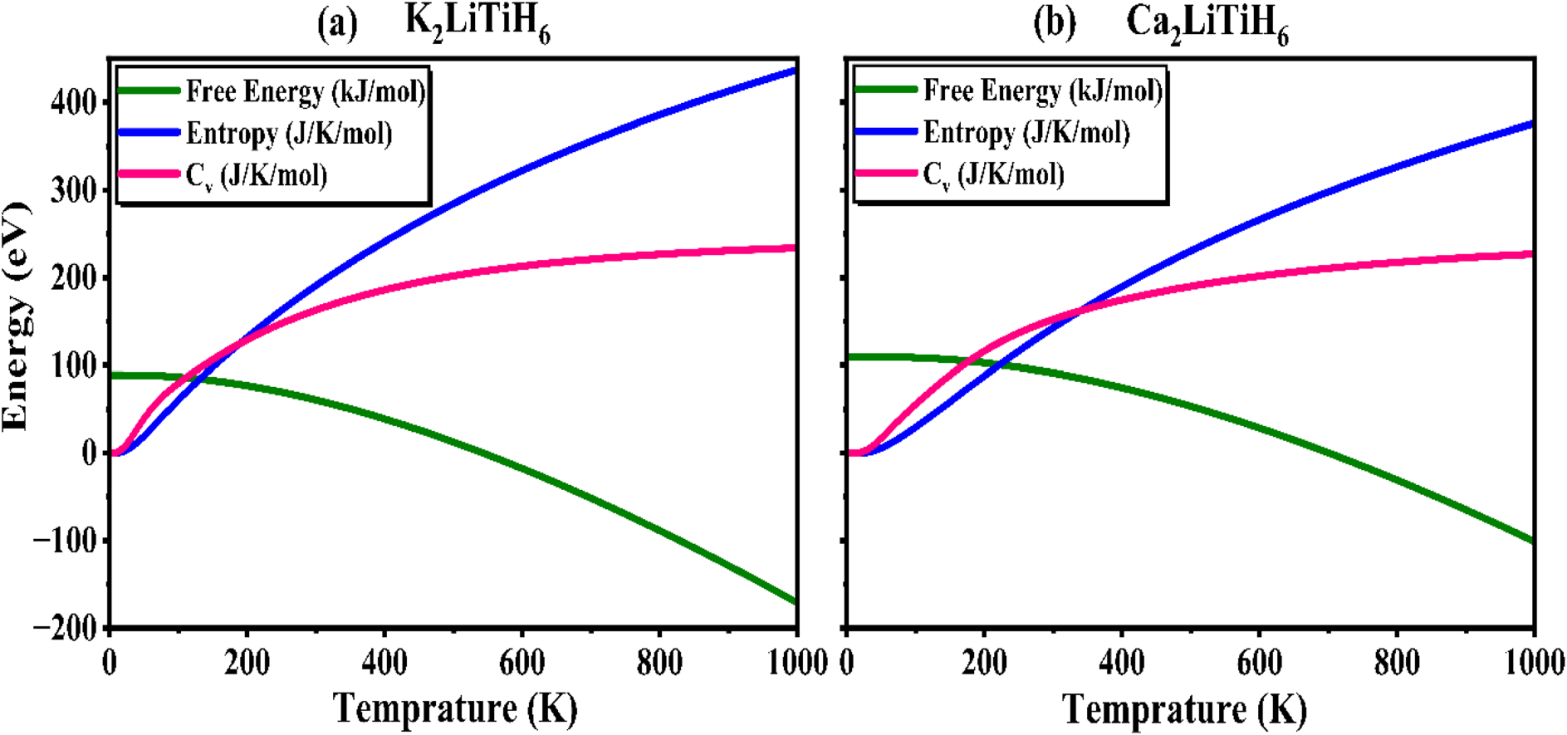
Variation in free energy, entropy change, and specific heat capacity with increasing temperature for (a) K_2_LiTiH_6_ and (b) Ca_2_LiTiH_6_.

In addition, thermodynamic properties such as Helmholtz free energy, entropy, and heat capacity, also calculated (presented in Fig. 8) to support the claim that these materials are suitable for hydrogen storage. Helmholtz free energy is an integral component in evaluating the thermodynamic favorability of hydrogen desorption at different temperatures. Hydrogen release at elevated temperatures is thermodynamically more advantageous when the free energy is negative. While the free energy of K_2_LiTiH_6_ and Ca_2_LiTiH_6_ increases with temperature, Ca_2_LiTiH_6_ exhibits a more stable hydride phase at higher temperatures, suggesting that it would be able to release hydrogen more efficiently at high temperatures. The entropy of the system represents the degree of disorder, and its variation with temperature provides insight into the process of hydrogen release in the material. According to our observations, both materials exhibit an increase in entropy with increasing temperature, indicating a growing disorder as hydrogen is released. Due to the larger entropy change in Ca_2_LiTiH_6_ at elevated temperatures, it may be more efficient at desorbing hydrogen at higher temperatures since the transition from the hydride phase to the free hydrogen phase increases the randomness of the system. The high entropy in hydrogen storage systems facilitates efficient hydrogen desorption, a crucial factor in optimizing these systems.

Moreover, the heat capacity (*C*_v_) of a material is reflected in the degree to which it can absorb heat during the process of hydrogen absorption or desorption. As the temperature increases, both K_2_LiTiH_6_ and Ca_2_LiTiH_6_ have increased *C*_v_, indicating that they are capable of absorbing thermal energy. It is interesting to note that the higher heat capacity observed for Ca_2_LiTiH_6_ suggests that it may be able to store more thermal energy, which may be beneficial for maintaining temperature stability during hydrogen release. It is crucial to prevent thermal runaway and ensure that the system operates within safe temperature limits. Finally, after assessing thermodynamic behavior, Ca_2_LiTiH_6_ provides superior thermal stability, hydrogen desorption efficiency, and thermal management compared to K_2_LiTiH_6_. The properties of Ca_2_LiTiH_6_ make it a more promising candidate for high-temperature hydrogen storage applications, while K_2_LiTiH_6_ may still be suitable for low-temperature hydrogen storage applications. The insights gained from these results ensure that both materials can be optimized for reliable and efficient hydrogen storage systems, contributing to the development of next-generation energy storage solutions.

## 4. Conclusion

This study establishes A_2_LiTiH_6_ (A = K, Ca) double perovskite hydrides as thermodynamically stable candidates for solid-state hydrogen storage. The compounds crystallize to form a cubic Fm-3m structure and have negative formation energies and favorable tolerance factors, indicating that they are both structurally and thermodynamically stable. A comparative analysis of A-site substitution reveals that Ca-based hydride possess shorter lattice constans, higher density, resulting in proved grvimetric capacity (∼4.38%), and lower desorption temperatures (∼380 K). As a sreuslt of these characteristics, Ca_2_LiTiH_6_ is particularly promising for releasing hydrogen under moderate conditions with high efficieny. With a higher lattice constant and lower density, K_2_LiTiH_6_ has hydrogen storage capacity (4.29 wt%), however it still demonstrates thermodynamic stability and may be useful for off-board storage applications. Both compounds exhibit elastic stability, with Ca_2_LiTiH_6_ exhibiting higher bulk and shear moduli, indicating a higher degree of rigidity and thermal resilience compared to K-based systems. There is evidence of metallic-like behavior in both compounds, with a slight Fermi-level overlap that enhances charge transfer and hydrogen desorption kinetics. Ca_2_LiTiH_6_ is more dielectrically sensitive and has a higher optical conductivity, which underscores its potential for use in multifunctional energy applications. These results highlight the critical role that substitution of A-sites (K *vs.* Ca) can play in customizing the structural, hydrogen storage, and thermodynamic properties of double perovskite hydrides. Despite the promise of both compounds, Ca_2_LiTiH_6_ appears to be a more suitable candidate for next-generation hydrogen storage and energy-related technologies. In the future, experimental validation will provide more information about their potential practical applications. Beyond lattice hydrogen storage in TiH_6_ octahedra, future work should examine adsorption on alternative sites such as A-site vacancies, interstitials, or defect states. These defect-mediated mechanisms might offer additional strategies for improving storage capacity and desorption characteristics.

## Author contribution

All authors verify their significant contribution to the work, and they equally accept public accountability for its content, from conceptual design to analytical work and writing and revising activities.

## Conflicts of interest

The authors declare that they have no financial relationships that could affect their research or personal relationships that could influence the information presented in this paper.

## Data Availability

All data in this study generated using the CASTEP code and available from the corresponding author on demand.
